# Supplementary material for: Prospects of Genomic Prediction in the USDA Soybean Germplasm Collection: Historical Data Creates Robust Models for Enhancing Selection of Accessions
Source: G3 (Bethesda). 2016 May 31;6(8):2329–41. doi: 10.1534/g3.116.031443 (PMC4978888; doi:10.1534/g3.116.031443)
Supplement: Supplemental Material [file supp_6_8_2329__index.html]

Prospects of Genomic Prediction in the USDA Soybean Germplasm Collection: Historical Data Creates Robust Models for Enhancing Selection of Accessions — Supplemental Material 

# Prospects of Genomic Prediction in the USDA Soybean Germplasm Collection: Historical Data Creates Robust Models for Enhancing Selection of Accessions

## Supplemental Material for Jarquin, Specht, and Lorenz, 2016

**Files in this Data Supplement:**

- Figure S1 - Exploration of the optimal number of genetic subpopulations (K) within the set of soybean accessions included in this study. (.pdf, 186 KB)
- Table S1 - Predictive abilities from the *One/Group*, *Group/All*, and *One/All* cross validation schemes for oil, protein, and yield in each trial using the G-BLUP model. (.xlsx, 13 KB)
- Table S2 - Predictive ability for seed yield using the *Group/All*, *One/All*, and *One/Group* cross validation schemes. (.xlsx, 13 KB)
- Table S3 - Predictions for *Group/Group* cross validation scheme with data grouped by trial. (.xlsx, 41 KB)
- Table S4 - Number and percentage of accessions belonging to each cluster separated by maturity group. (.xlsx, 13 KB)
- Table S5 - Raw phenotypes, corrected phenotyped, and predictions of phenotyped accessions comprising this study. (.xlsx, 4 MB)
- Table S6 - Genomic predictions (G-BLUP) of non-phenotyped accessions contained within the USDA Soybean Germplasm Collection. (.xlsx, 140 KB)
