# Supplementary material for: Prospects of Genomic Prediction in the USDA Soybean Germplasm Collection: Historical Data Creates Robust Models for Enhancing Selection of Accessions
Source: G3 (Bethesda). 2016 May 31;6(8):2329–41. doi: 10.1534/g3.116.031443 (PMC4978888; doi:10.1534/g3.116.031443)
Supplement: Supplemental Material [file supp_g3.116.031443_FigureS1.pdf]

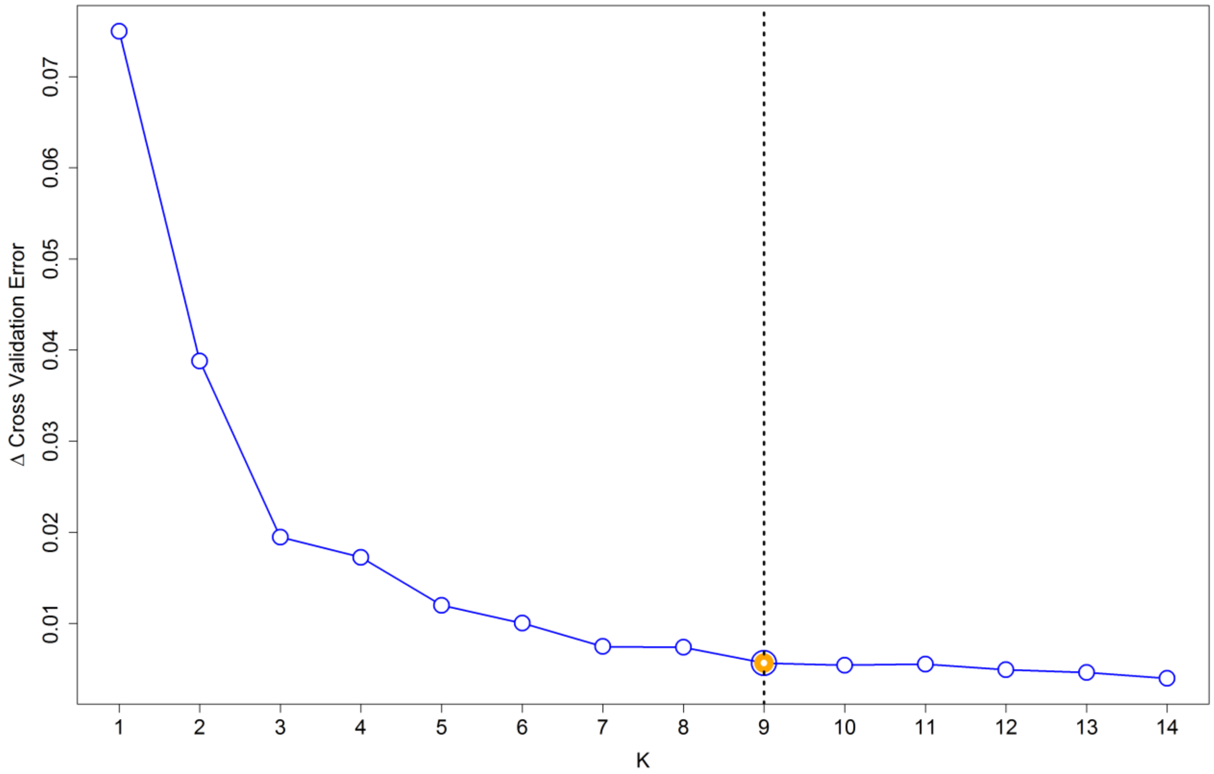

Figure S1. Exploration of the optimal number of genetic subpopulations (K) within the set of soybean accessions included in this study. A difference in cross-validation error between levels of K was used as a criteria. A plateau in Δ cross-validation error at K=9 was used to infer K.
